# Investigation of the Effects of the Multiplication Area Shape on the Operational Parameters of InGaAs/InAlAs SPADs

**DOI:** 10.3390/s26041228

**Published:** 2026-02-13

**Authors:** Anton Losev, Alexandr Filyaev, Vladimir Zavodilenko, Fedor Knyazhev, Igor Pavlov, Alexander Gorbatsevich

**Affiliations:** 1 “QRate” LLC, 2nd Donskoy Proyezd, 9/3, Moscow 115419, Russia; a.losev@goqrate.com (A.L.); v.zavodilenko@goqrate.com (V.Z.); f.knyazhev@goqrate.com (F.K.); ip@goqrate.com (I.P.); 2 NTI Center for Quantum Communications, National University of Science and Technology “MISIS”, Leninsky Prospect, 4/1, Moscow 119049, Russia; 3 Moscow Institute of Electronics and Mathematics, HSE University, Myasnitskaya Street, 20, Moscow 101000, Russia; 4 Department of Electronics and Nanoelectronics, National Research University “MPEI”, Krasnokazarmennaya Street, 14/1, Moscow 111250, Russia; 5 P.N. Lebedev Physical Institute of the Russian Academy of Sciences, Leninsky Prospect, 53, Moscow 119991, Russia; gorbatsevichaa@lebedev.ru; 6 Department of Quantum Physics and Nanoelectronics, National Research University of Electronic Technology “MIET”, Shokin Square, 1, Zelenograd 124498, Russia

**Keywords:** single-photon avalanche diode, single-photon detector, numerical simulation, InGaAs/InAlAs structure

## Abstract

A 2D model of an InGaAs/InAlAs single-photon avalanche photodiode has been developed. The influence of the active area structure in the multiplication region on the diode’s operating parameters has been studied. It was found that changing the diameter of the structure’s active region leads to a change in the dark current in the linear part of the current–voltage curve and a change in the breakdown voltage. Reducing the diameter of the active region from 25 μm to 10 μm allowed decreasing the dark current in the linear mode by about 10 dB. It has been shown that the quality of the SPAD device can be assessed by knowing the avalanche breakdown voltage and the overall current–voltage curve plot if we consider structures with the same multiplication region thickness and different remaining layers. The higher the breakdown voltage, the better the structure’s quality due to smaller local increases in the field strength. Following this statement, we conclude that for further use in single-photon detectors, it is reasonable to pick specific SPADs from a batch on the sole basis of their current–voltage curves.

## 1. Introduction

A single-photon avalanche photodiode (SPAD) for detecting emission at a wavelength of λ=1550 nm can be fabricated from different materials. For example, structures based on InGaAs/InP semiconductor materials have found widespread application [1,2,3,4,5,6,7,8,9,10]. Diodes based on this material pair demonstrate superior values of essential operational characteristics: high photon detection efficiency (PDE), low dark count rate (DCR), small afterpulse probability (AP), and relatively low dead time (DT).

However, the scientific community continues to develop SPADs based on other material pairs, such as Si/Ge [11,12,13,14,15] and InGaAs/InAlAs [16,17,18,19,20]. In this paper, we study an InGaAs/InAlAs structure.

The feature of SPAD based on InGaAs/InAlAs is that the avalanche generation process is excited by a single electron, unlike the InGaAs/InP structure, where it is triggered by a hole. Accordingly, the avalanche process is mainly supported by the represented types of carriers in the avalanche [21,22].

The advantage of using InAlAs material as a multiplication region is the high electron mobility, which allows much quicker quenching of the excited avalanche and bringing the structure to its equilibrium state. As a result, this device can have a higher cut-off frequency and better afterpulse characteristics than a device with an InP multiplication region. This is because the hole mobility in InP is lower than the electron mobility in InAlAs [23].

Nevertheless, commercially available SPAD devices for λ=1550 nm are mainly built on InGaAs/InP materials. The main obstacle to the fabrication of SPADs based on InGaAs/InAlAs materials is a large number of defects in the InAlAs material, which does not allow realizing its full potential [24,25,26]. Improvements in the growth process of this material will enable competing with these two types of devices. This study does not address the fabrication process of such structures, as it remains within the scope of theoretical modeling. The presented structures are considered laboratory prototypes and are not currently part of any production pipeline.

The simulated InGaAs/InAlAs SPAD structure is based on a design realized in works such as [26]. It should be noted that this structure, due to the inherent properties of the absorption layer material (InGaAs), is prone to high intrinsic defect and impurity concentrations. This leads to elevated DCR and high AP, which are primary noise sources that limit the overall detector performance. Consequently, such non-optimized structures often exhibit characteristics primarily suitable for laboratory research and proof-of-concept demonstrations, rather than for serial commercial applications. Such devices are typically fabricated using molecular beam epitaxy (MBE) or metalorganic chemical vapor deposition (MOCVD) to grow the heterostructure. Subsequent processing involves standard photolithography, wet or dry etching for mesa isolation, and dielectric deposition for passivation and anti-reflection coating. This combination of epitaxial growth and planar processing is standard for III-V compound SPADs.

In addition, the optimal sequence of heterojunction layers and their parameters are likely to differ significantly between InGaAs/InP- and InGaAs/InAlAs-based devices. Structure optimization, which is addressed in this work, is the second vital task to unlock the potential of the InGaAs/InAlAs device.

In this work, the effect of the shape of the multiplication region on the electric field strength distribution and the impact ionization in the structure has been investigated. The current–voltage characteristic (CVC) plots for different types of structures were also compared.

## 2. Two-Dimensional Modeling of InGaAs/InAlAs SPADs Structure

The modeling was performed in the T-CAD system. Based on the referenced study, our model builds upon the structure described by [26]. The model implements a comprehensive physics suite for recombination analysis. For carrier recombination and generation, it actively employs the Shockley–Read–Hall (SRH) model with doping and temperature dependence for trap-mediated processes, the standard Auger model for high-injection effects, and the Radiative model for band-to-band photon emission. Crucially, for avalanche photodiode operation, it integrates the field-dependent Avalanche model (van Overstraeten) to calculate the impact ionization rates, which is the core mechanism for internal gain. Furthermore, it includes a detailed Optical Generation module with ray tracing to simulate the photogeneration of carriers from an incident 1550 nm light source, enabling the study of photoresponse. The finite element modeling solved a system of continuity equations for electrons and holes and Poisson’s equation.

The simulation utilizes the validated material parameters from the integrated T-CAD library. This approach ensures the use of datasets for critical properties such as bandgap, carrier mobilities, and recombination coefficients in the InGaAs/InAlAs material system.

While the explicit modeling of discrete deep-level defects (traps) is not activated in this configuration, their primary physical effect on carrier dynamics—an increase in SRH recombination—is inherently captured and can be precisely controlled. The model’s active SRH recombination parameters (lifetime, doping dependence) provide a direct and effective mechanism to account for the influence of deep centers on device characteristics by adjusting the effective carrier lifetimes accordingly.

In order to limit the active region of the multiplication region (the region where the main avalanche generation process takes place), it is necessary to use different widths in the active and inactive multiplication regions. In the active region, we need to achieve a higher field strength; so, the width of the forbidden region should be smaller. The following problems arise when implementing this principle of building an active region.

- One has to arrange a transition between the two widths of the multiplication region. This can be achieved using either a sharp or a smooth transition. However, any sharp transition will result in a local increase in intensity and, therefore, a significant increase in the dark count rate when the instrument is operating in Geiger mode.
- More than one transition can be used to minimize the field strength in the inactive region. This means that not only can two different multiplication region widths be used, but three or more. This solution enables a significant reduction in the volume of high electric field areas in the inactive region and, consequently, a substantial decrease in the dark count rate when the device is operated in Geiger mode.

In this paper, three SPAD structures with different shapes of the multiplication region have been proposed. The first structure had two levels of the multiplication region and a sharp transition (2 lvls sharp), the second one had three levels of the multiplication region and a sharp transition (3 lvls sharp), and the third one had three levels of the multiplication region and a smooth transition (3 lvls smooth). The width of the multiplication region in the active region of all structures was the same: 0.8 μm. The width of the buffer 2 region in the active region was also the same: 1.8 μm. The thickness of each level of the multiplication region was 0.6 μm.

In the simulated structure, the diameter of the active region is 25 μm. The diameter of the whole simulated structure was 45 μm.

In the following subsections, the profiles of the electric field strength distribution and the impact ionization in each of the described structures are considered.

### 2.1. Two-Level Multiplication Region with a Sharp Transition

**Figure 1:**
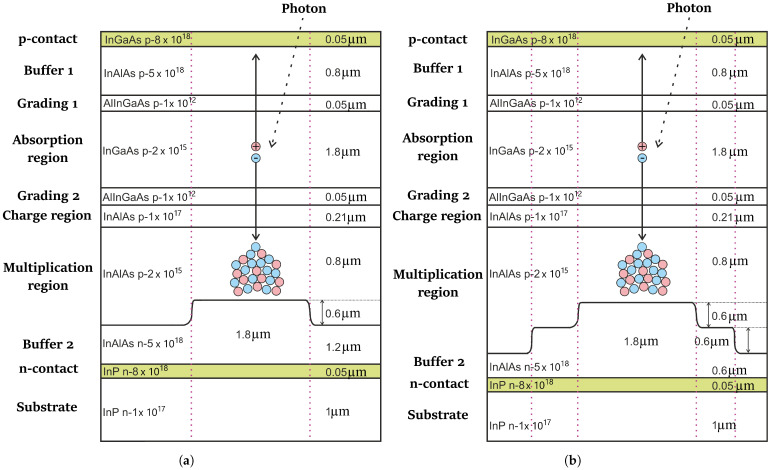
(**a**) Schematic representation of a 2D SPAD structure with a two-level multiplication region with a sharp transition. (**b**) Schematic representation of a 2D SPAD structure with a three-level multiplication region with a sharp transition.

A structure with two levels of multiplication region with a sharp transition is shown in Figure 1a. The dotted line separates the active and inactive regions.

**Figure 2:**
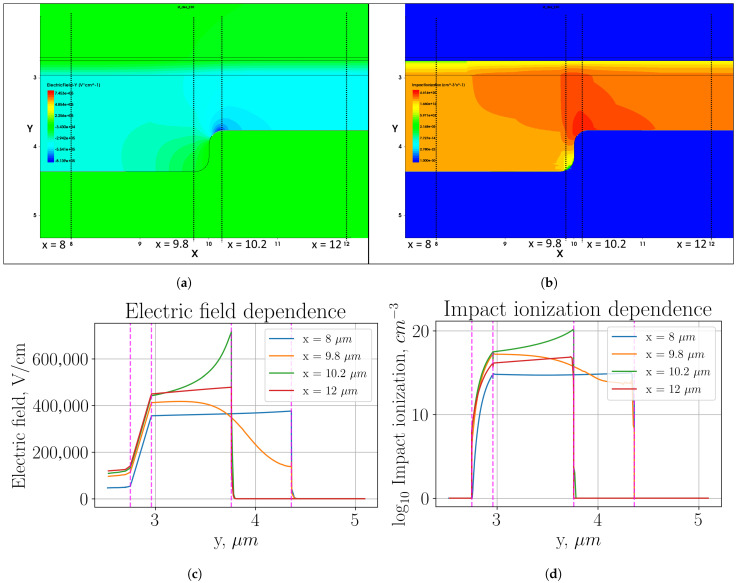
Two-level multiplication region with sharp transition: (**a**) heat map of the electric field strength distribution; (**b**) heat map of the impact ionization distribution; (**c**) electric field distribution profile in the cross-sections indicated in plot (**a**); (**d**) impact ionization distribution profile in the cross-sections indicated in plot (**b**).

Figure 2 shows heat maps of the electric field distribution and impact ionization (plots (a) and (b), respectively) in the multiplication region, as well as profiles of these parameter distributions in individual sections (plots (c) and (d), respectively).

The disadvantage of using a sharp transition is a large local increase in the electric field strength, as shown in plot (c). For the section x=10.2μm, the electric field strength increased to values of $E_{loc-enh}$ = 700 kV/cm, which is 40% stronger than the field in the remaining active region $E_{act}$ = 500 kV/cm. This field increase led to an increase in the impact ionization in this local area of about 60 dB. Since the size of this region is about 0.5 μm, we can estimate the contribution of this detriment to the overall DCR. The ring area where this effect occurs can be calculated as $S_{loc-enh}=\pi /4\;\left(D_{loc-enh+}^{2}-D_{loc-enh-}^{2}\right)=0.785*\left(25.5^{2}-24.5^{2}\right)\approx 40\;\mu$m^2^. It takes about 10% of the active area. Accordingly, the fraction of the dark counts generated by the local increase in field strength will be 40 dB higher than in the active region. It is, therefore, more appropriate to focus development efforts directly on addressing this drawback.

In the next section, where three multiplication regions with sharp transitions are considered, an attempt is made to reduce the DCR by reducing the field strength in the inactive region and hence the impact ionization.

### 2.2. Three-Level Multiplication Region with a Sharp Transition

A structure with a three-level multiplication region with a sharp transition is shown in Figure 1b. The dotted line separates the active and inactive regions.

**Figure 3:**
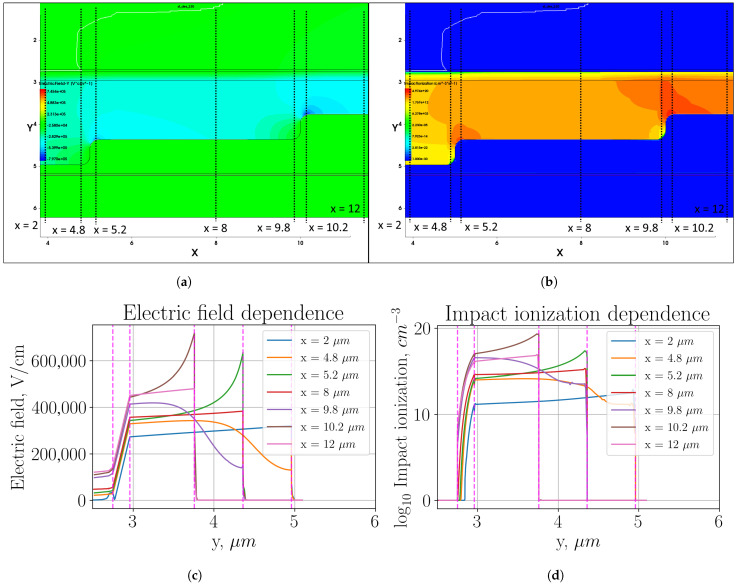
Three-level multiplication region with sharp transition: (**a**) heat map of the electric field strength distribution; (**b**) heat map of the impact ionization distribution; (**c**) electric field distribution profile in the cross sections indicated in plot (**a**); (**d**) impact ionization distribution profile in the cross sections indicated in plot (**b**).

Figure 3 shows heat maps of electric field distribution and impact ionization (plots (a) and (b), respectively, in the multiplication region, as well as profiles of these parameter distributions in individual sections (plots (c) and (d), respectively).

The described optimization of the structure, on the contrary, has further aggravated the problem of the local increase in the electric field and the impact ionization because another ring has been added (the outer one), in which we have again seen the effect of the local increase in the parameters at the sharp transition. As can be seen in Figure 3c,d, the electric field strength in the second ring reaches 620 kV/cm, and the impact ionization is about 20 dB higher than in the active region. This value does not make as large an additional contribution to the total dark count rate as the first transition. However, as can be seen, in the thickest multiplication region (x=2μm), the impact ionization is about four orders of magnitude lower than in the active region. In the mid-thickness multiplication region, the impact ionization is about two orders of magnitude lower than in the active region.

Thus, the use of a three-level multiplication region structure is only justified if the problem of increased local field at sharp transitions can be solved.

### 2.3. Three-Level Multiplication Region with a Smooth Transition

**Figure 4:**
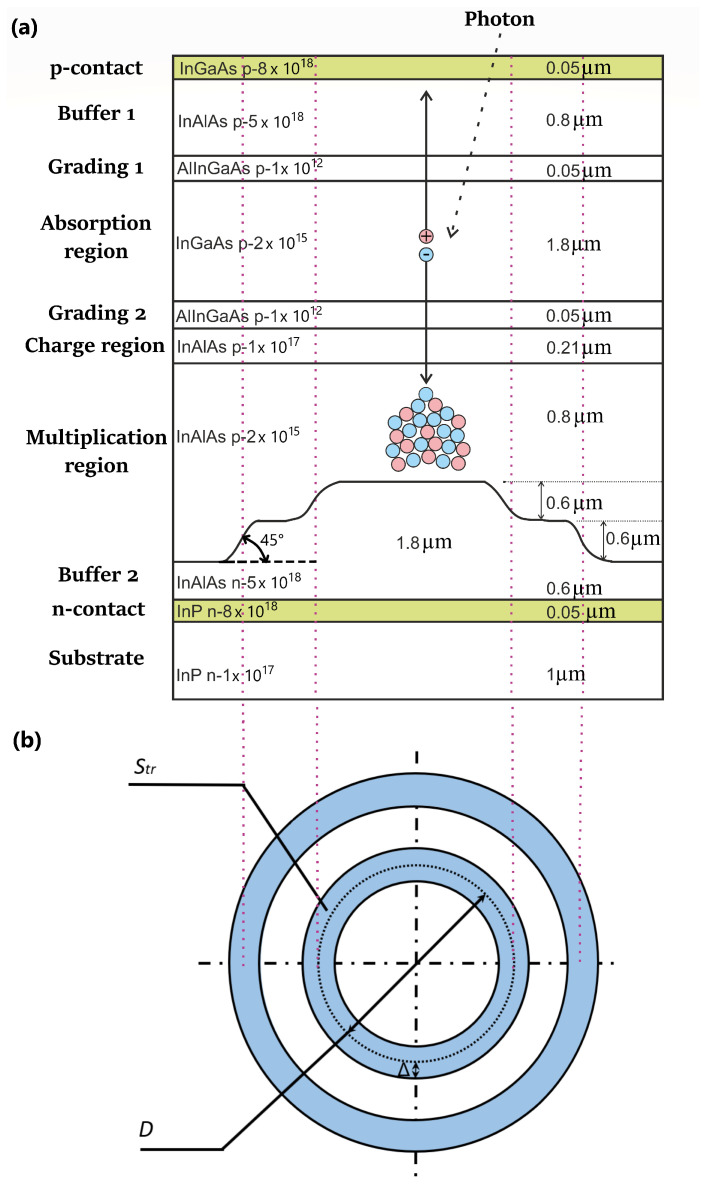
(**a**) Schematic representation of a 2D SPAD structure with a three-level multiplication region with smooth transition. (**b**) Schematic representation of smooth transitions in the multiplication area: Δ: half-width of the transition area; *D*: diameter of the active area, $S_{tr}$: area of the transition area.

A structure with three levels of a multiplication region with a smooth transition is shown in Figure 4. The dotted line separates the active and inactive regions.

**Figure 5:**
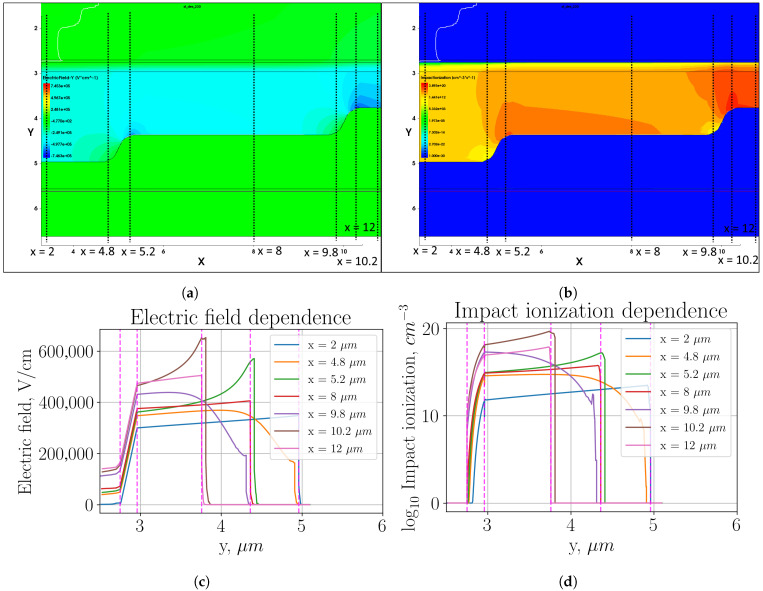
Three-level multiplication region with smooth transition: (**a**) heat map of the electric field strength distribution; (**b**) heat map of the impact ionization distribution; (**c**) electric field distribution profile in the cross sections indicated in plot (**a**); (**d**) impact ionization distribution profile in the cross sections indicated in plot (**b**).

Figure 5 shows heat maps of electric field distribution and impact ionization (plots (a) and (b), respectively) in the multiplication region, as well as profiles of these parameter distributions in individual sections (plots (c) and (d) respectively).

Using a smooth transition has reduced the electric field strength in the region of maximum local enhancement to 620 kV/cm from 700 kV/cm with a sharp transition. It has reduced the ratio of the impact ionization in the local enhancement region to that in the active region by two orders of magnitude. Thus, the dark count rate due to local enhancement is now only 20 dB DCR in the entire active region. The impact ionization in the second transition is approximately equal to the impact ionization in the active region, and, accordingly, the dark count contribution is approximately 10% of the dark count rate in the active region. The dark count rate was reduced by nearly the same amount by using a three-level multiplication region, as compared to the two-level region structure.

Thus, creating smooth transitions at the boundaries of the multiplication region levels is an extremely effective method of reducing the dark count rate in the device as a whole. If one could achieve sufficiently smooth transitions (see Figure 4) in the design of the device so that the dark count rate contribution by the local enhancements is no more than 20 dB higher than in the active region of the device, then a three-level multiplication region can be considered for implementation.

**Figure 6:**
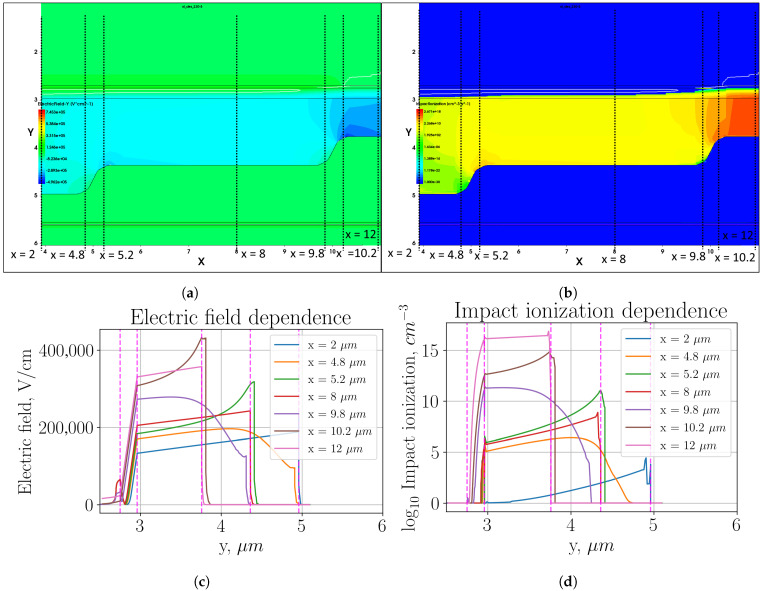
Three-level multiplication region structure with smooth transition with incident radiation intensity 10μW/cm^2^: (**a**) heat map of electric field strength distribution; (**b**) heat map of impact ionization distribution; (**c**) electric field distribution profile in the cross sections indicated in plot (**a**); (**d**) impact ionization distribution profile in the cross sections indicated in plot (**b**).

**Figure 7:**
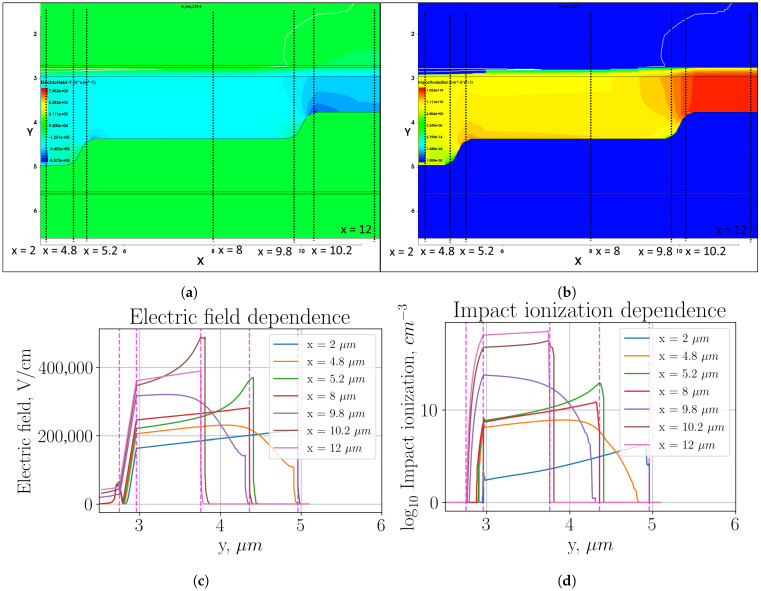
Three-level multiplication region structure with smooth transition with incident radiation intensity 100μW/cm^2^: (**a**) heat map of electric field strength distribution; (**b**) heat map of impact ionization distribution; (**c**) electric field distribution profile in the cross sections indicated in plot (**a**); (**d**) impact ionization distribution profile in the cross sections indicated in plot (**b**).

**Figure 8:**
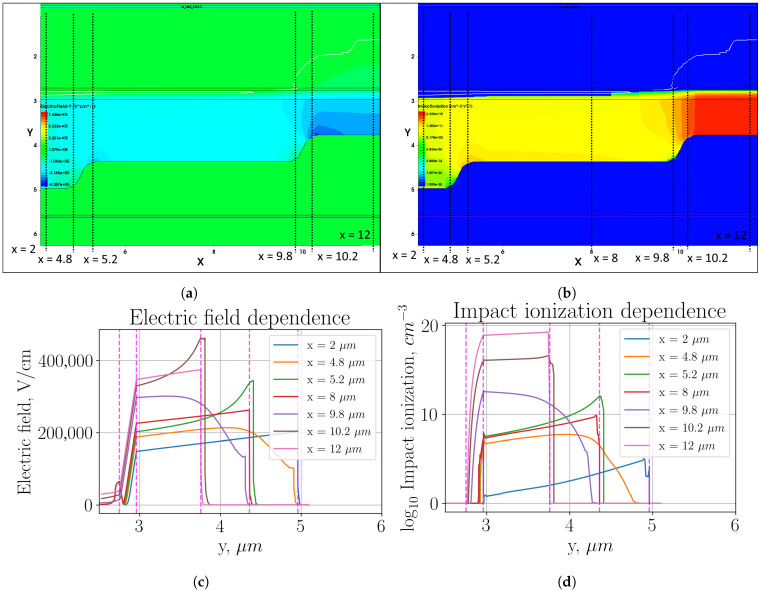
Three-level multiplication region structure with smooth transition with incident radiation intensity 1000μW/cm^2^: (**a**) heat map of electric field strength distribution; (**b**) heat map of impact ionization distribution; (**c**) electric field distribution profile in the cross sections indicated in plot (**a**); (**d**) impact ionization distribution profile in the cross sections indicated in plot (**b**).

Figure 6, Figure 7 and Figure 8 demonstrate electric field distribution and profiles and impact ionization for incident radiation with wavelength λ=1550 nm and intensities 10μW/cm^2^, 100μW/cm^2^, and 1000μW/cm^2^, respectively.

These figures were made for lower bias voltage than the plots without incident radiation. Therefore, the electric field strength in the multiplication region is much lower than in the previous plots.

To thoroughly investigate the impact ionization, the structure was fully irradiated, not just in the active region. The simulated structure assumes ideal contacts and does not include top metal or $SiO_{2}$ passivation in order to isolate the effects of the multiplication region geometry.

In all the figures shown, with structures illuminated in the active region, the impact ionization is significantly higher than the impact ionization in the region of local field enhancement. This observation is explained by the dominant role of SRH recombination in the local field enhancement region, where a high defect density promotes rapid carrier recombination before an avalanche process can develop. The impact ionization in the region of intermediate thickness of the multiplication region is lower by about eight orders of magnitude compared to the impact ionization in the active region for all considered incident radiation intensities.

This peculiarity of the considered structure allows applying it as a low noise avalanche photodiode (APD), i.e., operating it in linear mode.

The obtained distributions with incident radiation allow us to assess the peculiarities of the structure operation only in the linear mode. When operating in single-photon mode, only the curves without incident radiation must be considered.

### 2.4. Simulation of Devices with Different Active Area Diameters

In this stage of the simulation, structures with three levels of multiplication region with a smooth transition were compared. The structures described above had an active region diameter of $D_{act}^{\left(25\right)}=25\;\;\mu$m. Next, we consider the structures with diameters $D_{act}^{\left(15\right)}=15$ and $D_{act}^{\left(10\right)}=10\;\;\mu$m.

**Figure 9:**
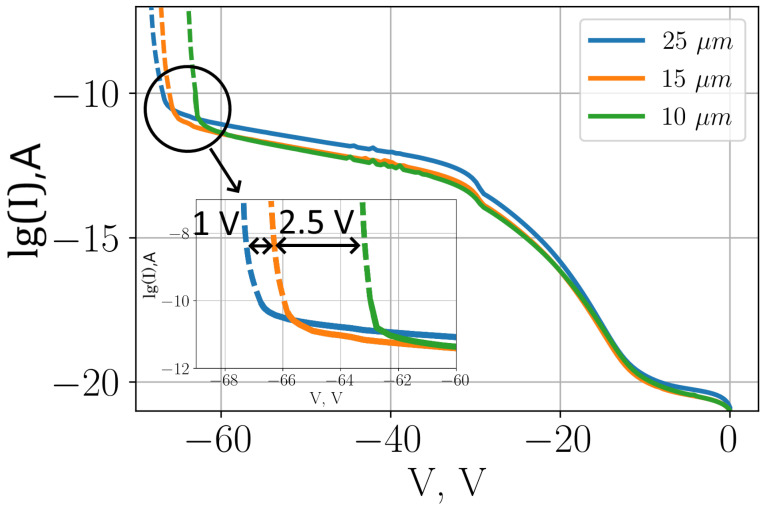
Comparison of the dark CVC for SPADs with a three-level structure of the multiplication region with smooth transitions for different diameters of the active region: D={25,15,10}μm.

The main advantage of using structures with a reduced active region diameter is a lower value of the dark current. On the other hand, the main drawback is the need for additional focusing of the radiation hitting the diode. In the simulation results presented, the CVCs of the dark current for diodes with different active region diameters have been compared (see Figure 9).

As we can see in this figure, devices with a smaller active region diameter have a lower dark current in the linear part of the CVC. However, these devices also have a lower breakdown voltage.

The dark current in the linear region of the 25μm structure is higher than that of the 10μm structure by approximately a factor of 3. The dark current in the linear region is nearly the same for the 15μm and 10μm diameter structures.

The breakdown voltage for the 25μm diameter active region is 1 V higher than the value for the 15μm diameter structure, which in turn is 2.5 V higher than the breakdown voltage of the 10μm diameter structure.

Let us consider the mechanism of dark current reduction in the linear region of the CVC.

The value of the active area for the discussed structures can be calculated by the circle area formula.

Thus, the expected reduction in the level of dark current in the active region should be proportional to the area of the active region. However, a larger contribution to the dark current is made at transitions between levels of the multiplication region. The contribution of this quantity is proportional to the diameter of the active region. Assuming that the half-width of the transition region is Δ=0.5μm, we can calculate the transition area as follows: $S_{tr}=\frac{\pi }{4}\left({\left(D+\Delta \right)}^{2}-{\left(D-\Delta \right)}^{2}\right)=\pi D\Delta$.

We can also calculate the relative area as $s_{rel}=\left(S_{tr}\right)/\left(S_{act}\right)=\left(\pi D\Delta \right)/\left(\frac{\pi }{4}D^{2}\right)=4\;\Delta /D$.

- 25 μm: $S_{act}^{\left(25\right)}\approx 490.9\;$${\mu m}^{2}$→$S_{tr}^{\left(25\right)}\approx 39.3\;$${\mu m}^{2}$ → $s_{rel}^{25}\approx 0.08=8\%$
- 15 μm: $S_{act}^{\left(15\right)}\approx 176.7\;$${\mu m}^{2}$ → $S_{tr}^{\left(15\right)}\approx 23.6\;$${\mu m}^{2}$ → $s_{rel}^{15}\approx 0.013=13\%$
- 10 μm: $S_{act}^{\left(10\right)}\approx 78.5\;$${\mu m}^{2}$→$S_{tr}^{\left(10\right)}\approx 15.7\;$${\mu m}^{2}$→$s_{rel}^{10}\approx 0.2=20\%$

The general equation for the dark current $I_{dcr}$ for this structure can be written as

(1) $$I_{dcr}=S_{act}j_{act}+S_{tr}j_{tr},$$

where $j_{act}$ is the current density in the active region; $j_{tr}$ is the current density in the transition region.

Given that $S_{act}$ is related to $S_{tr}$, this equation can be rewritten as

(2) $$I_{dcr}=j_{act}\frac{\pi }{4}\left(D^{2}+4kD\Delta \right)=j_{act}\frac{\pi }{4}D^{2}\left(1+4k\frac{\Delta }{D}\right).$$

**Figure 10:**
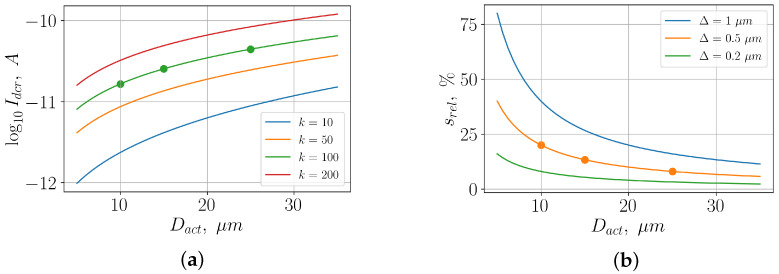
(**a**) Dependence of the dark current on the diameter of the active region for different ratios of the current density in the transition region and the active region. (**b**) Dependence of the relative area $s_{rel}$ on the diameter of the active region at different values on the width of the transition region in the multiplication region.

Thus, the larger the ratio $\frac{\Delta }{D}$, i.e., the smaller the diameter *D* for a given Δ, the larger the influence of the transition region on the dark current. Let us assume that the current density $j_{act}$ in the active region does not depend on the area of the active region. The dependence of the dark current on the diode diameter, in this case, is shown in Figure 10a. The dots mark the device parameters used in the simulation.

We can see from these figures that, by reducing the diameter of the active region from 25μm to 10μm, we can reduce the dark current level by approximately 8.5 dB. In Figure 9, we consider the linear region of the CVC. We can see that the reduction in dark current is in good agreement with the theoretical predictions.

The reduction in breakdown voltage by a decrease in the diode diameter is related to changes in the relative sizes of the active and transition areas in the multiplication region (specifically, the increase in the relative area $s_{rel}$). An increase in $s_{rel}$ results in a larger relative fraction of the structure having a smaller electric field boundary for avalanche breakdown. Thus, the larger the relative area, the lower the breakdown voltage for the same value of the transition region width.

Figure 10b shows the dependence of $s_{rel}$ on the diameter of the active region for different values of the transition region width in the multiplication region. The points correspond to the parameters at which the simulation was performed.

A three-level multiplication region with smooth transitions provides a reduction in electric field inhomogeneities and, consequently, avalanche generation at the boundaries between the levels of the multiplication region. It should be noted that the formation of smooth transitions in the multiplication region is more technological than growing a multilevel structure using other approaches. In this design, the electric field is localized predominantly in the active region of the multiplication region, resulting in a lower dark current in the linear section of the CVC and DCR when operating the diode in the Geiger mode. This also means that decreasing the diameter of the active region of the multiplication region allows reducing the zone of the electric field localization responsible for avalanche generation, which leads to an even larger decrease in the DCR and dark current.

## 3. Results and Discussion

In this section, we compare the presented structure types based on the analysis of the CVC.

**Figure 11:**
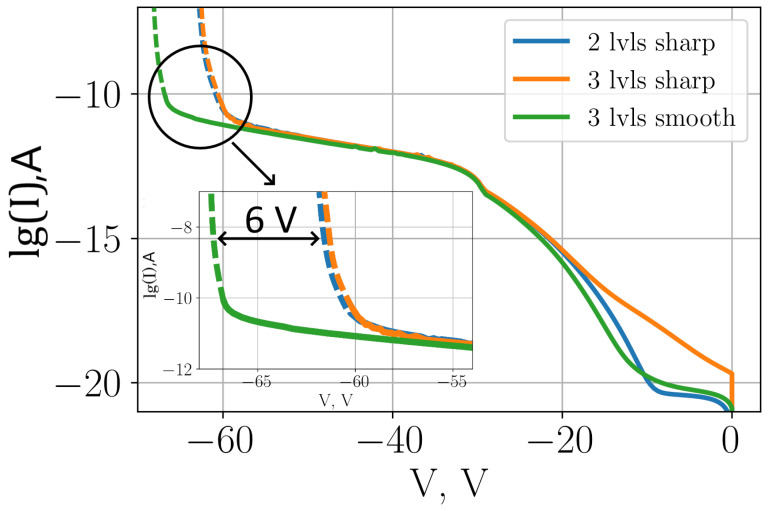
Comparison of the dark current for SPADs with different types of structures.

Figure 11 shows the CVCs for different types of structures without incident radiation. The avalanche breakdown for a structure with a three-level multiplication region with sharp transitions occurs about 200 mV earlier than for a structure with a two-level multiplication region with sharp transitions. The avalanche breakdown voltage fully determines the limits of device operation in Geiger and linear modes.

In the considered structures, avalanche breakdown occurs primarily in transitions between different levels of the multiplication region. Therefore, the higher the local voltage at the transition, the lower the breakdown voltage value of the structure. The plots of CVC and breakdown voltage quantitatively express the result of the combined effects of generation and recombination and allow us to assess the quality of the created structure. The primary electron (photon- or noise-generated) in the active region of the structure will have an extremely low probability of generating an avalanche process, because the local breakdown voltage in the active region of the structure will be much higher than that applied to the SPAD. However, by all operation indicators, it will appear to work in the Geiger mode, due to avalanche breakdown in transitions between the multiplication region levels. Such a device would, therefore, have an extremely low PDE. We can conclude that the smaller the local increase in field strength in the transitions, the higher PDE the SPAD would have in operation. This effect can be diagnosed by the CVC of the device.

The 6 V difference between the breakdown voltage values of the considered structures clearly shows that the PDE of the SPAD based on the structure with three levels of the multiplication region and a smooth transition (see Figure 4) will be significantly higher than that of the SPAD based on the other presented structures. This once again proves that the creation of smooth transitions between different levels of the multiplication region is an important aspect of SPAD structure optimization.

**Figure 12:**
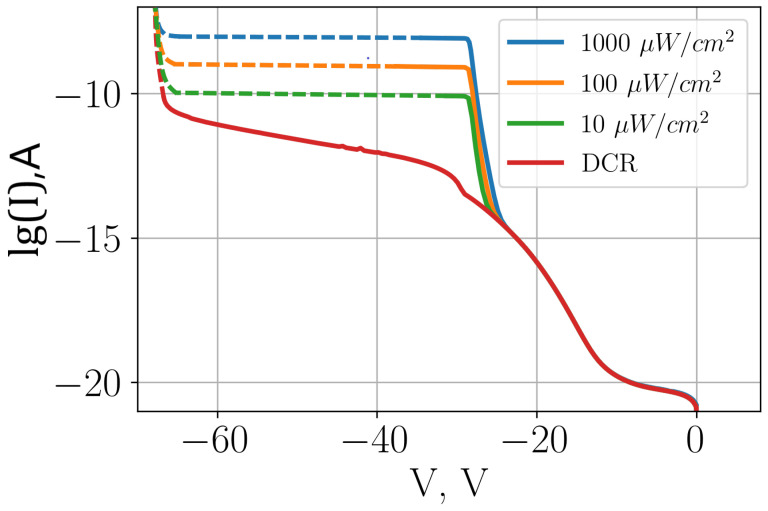
Comparison of CVC for different incident radiation intensities for SPADs with a three-level multiplication structure with smooth transitions.

Figure 12 shows CVC plots for different incident radiation intensities for a SPAD with a three-level multiplication structure with a smooth transition. This figure shows that an order of magnitude change in incident radiation intensity results in order of magnitude change in current, at least in the range of 10–1000 μW/cm^2^. Thus, the device based on the structure presented in this paper can be effectively used not only in the Geiger mode but also in the linear mode, such as the APD.

To substantiate the relevance of the problem we are investigating, we can refer to the experimental work by [27]. In that study, InAlAs/InGaAs avalanche photodetectors—structures conceptually similar to those we model—were experimentally characterized. However, it should be noted that experimental data on dark currents for InGaAs/InAlAs-based SPADs are currently limited, due to the technological challenges related to the quality of the InAlAs material. Our simulation results should, therefore, be considered in the context of a theoretical study aimed at optimizing the multiplication region geometry. Direct comparison with experimental data will become more feasible as epitaxial growth technologies improve.

## 4. Conclusions

The simulation and analysis of electric field profiles and impact ionization in different structures have shown that the creation of smooth transitions at the level boundaries of the multiplication region is an extremely effective method of DCR reduction in SPAD. If one manages to achieve sufficiently smooth transitions (see Figure 4) in the device design so that the dark count rate increase due to local field enhancement does not exceed 20 dB above the active region value, a three-level multiplication region can be considered for implementation.

It has been shown that changing the diameter of the active region of the device results in both a change in the dark current in the linear portion of the CVC and a change in the breakdown voltage. It has been shown that decreasing the diameter from 25μm to 10μm reduces the dark current in the linear operation mode of the device by approximately 10 dB. However, it results in a breakdown voltage decrease by approximately 3.5 V. The first property leads to a decrease in DCR when the device is operated in the Geiger mode. However, the second property reduces the probability of photon detection.

It has been shown that the quality of the SPAD device can be assessed by the avalanche breakdown voltage and the overall CVC plot, provided the examined structures have equal thicknesses of the multiplication region and other layers of the structure. The higher the breakdown voltage, the better the structure’s quality, due to smaller local increases in the field strength. Following this statement, we conclude that for further use in single-photon detectors, it is reasonable to pick specific SPADs from a batch on the sole basis of their current–voltage curves.

The developed SPAD structure with three levels of multiplication region and smooth transitions can also be used for APD devices operating in linear mode. Such a device has the following property: when the intensity increases by one order of magnitude, the current changes by one order of magnitude as well, which is beneficial for the linearity of the characteristic. In addition, this device is characterized by low dark current and, therefore, a low signal-to-noise ratio.

An important part of our work is devoted to a dark current, which is a key factor influencing the DCR. Specific DCR values are not provided, as they pertain to Geiger-mode operation, whereas our calculations are performed for the linear regime. Correlating these two regimes would require a separate experimental investigation.

The obtained research results hold significant value for industrial single-photon avalanche photodiodes based on InGaAs/InP. By demonstrating how optimizing the multiplication region geometry—specifically, implementing smooth transitions and reducing the active area diameter—effectively reduces the dark current and dark count rate and increases the breakdown voltage, this study provides practical engineering principles that can be adapted and applied to enhance the performance and manufacturability of commercial InGaAs/InP SPAD devices.

## Data Availability

The data presented in this study are available upon request from the corresponding author.
